# Associated Cardiac and Extracardiac Anomalies in Patients with Abnormal Coronary Artery from the Pulmonary Artery

**DOI:** 10.1007/s00246-024-03760-x

**Published:** 2025-01-08

**Authors:** Pinar Bambul Heck, Franziska Ziermann, Andreas Simmelbauer, Maria von Stumm, Hazer Ercan Bozyer, Peter Ewert, Alfred Hager

**Affiliations:** 1 Department of Pediatric Cardiology and Congenital Heart Disease, Deutsches Herzzentrum München (DHM), Technische Universität München (TUM), Lazarettstr. 36, 80636 Munich, Germany; 2 Department of Congenital and Pediatric Heart Surgery, School of Medicine, German Heart Center Munich, University of Munich, TechnicalMunich, Germany; 3 European Kids Heart Centre, Ekhz, Munich, Germany; 4 Division of Congenital and Pediatric Heart Surgery, University Hospital of Munich, Ludwig-Maximilians-Universität, Munich, Germany; 5 Department of Pediatric Cardiology, Dokuz Eylul University Hospital, Izmir, Turkey

**Keywords:** Anomalous coronary artery from the pulmonary artery, Bland-White-Garland syndrome

## Abstract

Anomalous origin of coronary arteries from the pulmonary artery (ACAPA) are rare but clinically significant condition with high mortality if left untreated. Even more rarely, ACAPA is associated with other congenital heart defects. From 1974 to 2024, 120 patients with anomalous coronary arteries connected to the pulmonary artery were retrospectively analyzed. Medical records including surgical operative notes and angiography protocols were screened for any other cardiac and extracardiac defects. Anomalous left coronary artery connected to the pulmonary artery (ALCAPA) was present in 103 patients, anomalous right coronary artery connected to the pulmonary artery (ARCAPA) in 6, anomalous circumflex coronary artery connected to the pulmonary artery (ACXPA) in 7, anomalous left anterior descending coronary artery connected to the pulmonary artery (ALADPA) in 2, and anomalous single coronary artery connected to the pulmonary artery (ASCAPA) in 2 patients. Anomalous origin of the coronary artery from the pulmonary arteries was associated with other congenital heart defects in 16 patients (13%) and with extracardiac anomalies in 10 patients (8%). Most associated cardiac anomalies were left-sided obstructive defects or shunt-lesions. Patients with ACAPA and associated cardiac defects had poorer perioperative survival. A precise diagnosis of coronary anatomy is crucial for preoperative planning and the success of the surgery of patients with congenital heart defects. In particular, for patients with a challenging postoperative course, an anomalous coronary artery originating from the pulmonary artery should be considered.

## Introduction

Anomalous origin of coronary arteries from the pulmonary artery (ACAPA) is rare condition. Left untreated, it shows high mortality up to 90% due to cardiac ischemia and sudden cardiac death in relatively young patients [1–3]. The most common coronary anomaly with abnormal origin from the pulmonary artery is ALCAPA (anomalous left coronary artery from the pulmonary artery), also known as Bland-White-Garland syndrome [4]. It constitutes 0.3–0.5% of congenital heart defects [1, 2]. Abnormal origin of coronary arteries other than the left coronary artery is even less common and the literature is limited to case reports [5–7]. Since patients with abnormal origin of coronary arteries other than the left coronary artery don’t have the typical presentation of the more common ALCAPA with mitral valve regurgitation and impaired left ventricular function, timely clinical diagnosis is challenging. Management depends on clinical factors, though surgical reimplantation is often the treatment of choice [8].

The prognosis is associated with the degree of collateralization with the normal coronary artery and with the associated cardiac anomalies [9]. Coronary anomalies with abnormal origin from the pulmonary artery can be associated with other cardiac defects and often are misdiagnosed before surgery, mostly due to specific hemodynamics masking myocardial ischemia preoperatively. This is a further limiting factor for the prognosis and therefore crucial for further treatment.

This retrospective study aimed to determine the prevalence of associated cardiac anomalies in patients with abnormal coronary arteries from the pulmonary artery.

## Methods

### Study Population

All patients treated at the German Heart Center Munich from its opening in 1974 until June 2024, with anomalous coronary artery connections to the pulmonary artery, were included in this retrospective cohort study. Patients with coronary anomalies that didn’t drain into the pulmonary artery and those with coronary fistulas were excluded. A total of 120 patients were enrolled. Three patients had surgery at a different facility and were treated at our center for follow-up.

Data were retrospectively analyzed for all selected patients. Consent to analyze and publish data anonymously was present from all patients or the legal guardian, as appropriate. The study was in accordance with good clinical practice and the Declaration of Helsinki (version 2013).

### Data Collection

The database of pediatric cardiology at the German Heart Center Munich was searched for all patients with anomalous coronary arteries connected to the pulmonary artery. Medical reports, surgical records, echocardiographic data, and coronary angiography findings were examined for the presence of additional cardiac defects and extracardiac congenital anomalies as well as for assessing ventricular function and mitral valve regurgitation. Minor cardiac defects, such as patent foramen ovale (PFO) or a small coronary fistula from the right coronary artery to the right atrium, were not defined as associated cardiac defects. The exact anatomical origin of the anomalous coronary artery from the pulmonary artery (i.e., the pulmonary artery trunk, right pulmonary artery, and left pulmonary artery) was analyzed using operative notes or heart catheterization data for non-operated patients.

Perioperative mortality was defined as any death, regardless of cause, occurring within 30 days after surgery in or out of the hospital.

### Statistics

Statistical analysis was performed using SPSS software (version 23) for Windows (SPSS Inc., Illinois, USA), with a significance level of *α* < 5% (*p* < 0.05). Characteristics, frequencies and outcomes of patients with ACAPA are provided using descriptive statistics. Using cross-tabulation and Chi-square tests, the distribution of gender, various connection sites to the pulmonary artery or its branches, additional cardiac defects, isolated cases, and extracardiac anomalies were analyzed for the overall cohort and subgroups. Frequencies were also graphically represented using bar charts. Outcomes were compared between different ACAPA groups using Fisher’s exact test.

Graphical representation was done using box plots. Non-parametric tests were used due to the small sample sizes. The Kruskal–Wallis test assessed the location and the type of the anomaly in relation to the occurrence of associated cardiac defects, and extracardiac anomalies.

## Results

### Study Population

**Fig. 1:**
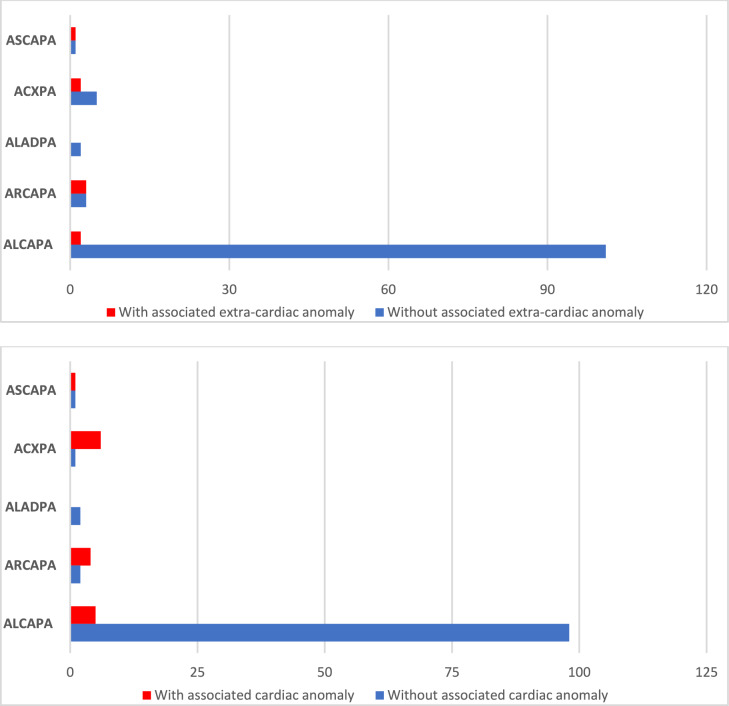
Overview of the distribution of associated cardiac and extra cardiac anomalies in patients with abnormal coronary artery from the pulmonary artery

A total of 120 patients were included in the study, comprising 77 female (64%) and 43 male (36%). The diagnostic groups within the cohort exhibit a highly uneven distribution: 103 patients with ALCAPA (86%), 6 with ARCAPA (5%), 2 with ALADPA (1.5%), 7 with ACXPA (6%), and 2 with ASCAPA (1.5%) (Fig. 1).

ACAPA was diagnosed prior to surgery in 115 patients. In two patients, the coronary anomaly was discovered during the initial cardiac surgery for other congenital heart defects (one patient with Shone complex and one patient with hypoplastic left heart syndrome—HLHS), in 3 patients it was diagnosed postoperatively via cardiac catheterization (2 patients with aortic coarctation and one patient with Scimitar syndrome). Six patients did not have a surgical repair. A conservative approach without surgical intervention was chosen for patients in advanced stages of the disease, considering the anticipated poor prognosis and advanced age. Also, the presence of adequate coronary collateralization and satisfactory ventricular function supported the decision for a conservative approach. Consequently, in two patients for whom a corrective operation was considered but ultimately not performed, both echocardiography and cardiac catheterization were conducted. One of these two patients exhibited an anomalous origin of the right coronary artery from the pulmonary artery, which, due to sufficient collateralization, resulted in only minimal impairment of right ventricular function.

### Pulmonary Artery Connection

There is a significant difference among the diagnostic groups regarding the location of the coronary artery connection to the pulmonary artery (*p* < 0.001). Notably, ACXPA patients frequently (6 out of 7 cases) have a connection to the right pulmonary artery. In total, 12 patients have a coronary artery connection to the right pulmonary artery, while only two patients have a connection to the left pulmonary artery. An overview of the location of the coronary artery connection to the pulmonary artery according to the subtypes is given in Table 1.

**Table 1:** An overview of the location of the coronary artery connection to the pulmonary artery according to the subtypes

	Study population *n* = 120	ALCAPA *n* = 103	ARCAPA *n* = 6	ALADPA *n* = 2	ACXPA *n* = 7	ASCAPA *n* = 2	p-value
PA	106	98	4	2	1	1	
RPA	12	3	2	0	6	1	
LPA	2	2	0	0	0	0	*p* < .001

#### Associated Cardiac Anomalies

Patients in the ARCAPA, ACXPA, and ASCAPA groups more frequently had associated cardiac defects (*p* = . < 001), compared to patients with ALCAPA, as shown in Fig. 1. Specifically, 5 of 103 ALCAPA patients (5%), 4 of 6 ARCAPA patients (67%), 6 of 7 ALCXPA (86%) patients and one of two ASCAPA patients had further congenital heart defect (Fig. 1).

Among patients with associated cardiac defects, left heart obstructions or shunt-lesions were most commonly observed. Only one patient had pulmonary valve stenosis, and there were no other right heart obstructions.

The accompanying cardiac defects included: Aortic coarctation (*n* = 8), bicuspid aortic valve (*n* = 2), ventricular septal defect (*n* = 6), atrial septal defects (*n* = 9), patent ductus arteriosus (PDA) (*n* = 5), aortic stenosis (*n* = 2 cases), aortic arch anomaly (*n* = 1), hypoplastic left heart syndrome (*n* = 2), left persistent superior vena cava (*n* = 3), aortopulmonary window (*n* = 1), Scimitar syndrome (*n* = 1), transposition of the great arteries (*n* = 1), pulmonary valve stenosis (*n* = 1). An overview of the individual associated cardiac anomalies is provided in Table 2.

**Table 2:** Overview of the patients with anomalous coronary arteries from pulmonary artery in relation to associated cardiac and extracardiac anomalies

Coronary anomaly	Localization PA	Associated cardiac defect	Associated non-cardiac defect	Surgery	Outcome	Tool of diagnostic	Time of the diagnosis
ALCAPA	PA	–	Pelvic kidney	–	Alive	Angiography	preoperative
ALCAPA	LPA	CoA, VSD, PDA	–	Resection of CoA, 2. Reimplantation of LCA	Died 1 day postoperatively	Angiography	postoperative
ALCAPA	PA	Truncus bicaroticus, PDA, LPSVC	Rubinstein-Taybi-Syndrom	–	Died 4 months after diagnosis	Angiography	preoperative
ALCAPA	PA	Scimitar Syndrom	Hypoplasia of the right lung	Repair Scimitar syndrome, reimplantation LCA	Lost to f/u	Angiography	preoperative
ALCAPA	PA	ASD II	–	ASD II closure, reimplantation LCA	Alive	Angiography	preoperative
ALCAPA	PA	ASD II, PDA	–	ASD II closure, ligation PDA reimplantation LCA	Alive	Echocardiography	preoperative
ALCAPA	RPA	Scimitar Syndrom, aortic coarctation	Hypoplasia of the right lung	Repair Scimitar syndrome, reimplantation LCA aortic arch repair	Died 2 weeks after surgery	Angiography	postoperative
ALPACA	PA	Pulmonary valve stenosis	–	Repair pulmonary valve stenosis, reimplantation LCA	Alive	Angiography	preoperative
ARCAPA	PA	ASD, Aortopulmonary window, LPVCS	Fetal alcohol syndrome	Closure of aortopulmonary window, Takeuchi repair, ASD suture, ligation of the left superior vena cava	Alive	Angiography	preoperative
ARCAPA	RPA	HLHS, PDA, PFO	–	Noorwood I, Reimplantation RCA	Died 4 months after surgery	Surgery	intraoperative
ARCAPA	PA	ASD, VSD, LPVCS	VACTERL association, hearing impairment	Reimplantation of RCA , ASD-Patch	Alive	Angiography	preoperative
ARCAPA	PA	TGA, VSD, CoA	Goldenhar-Syndrom	Arterial Swich, VSD patch closure, resection of CoA, reimplantation RCA	Alive	Echocardiography	Preoperative
ACXPA	RPA	CoA, VSD, PDA	–	Resection of CoA, Ligation PDA, VSD patch closure Second surgery: re-implantation LCX	Alive	Angiography	postoperative
ACXPA	RPA	HLHS	Pulmonary hypoplasia (missing upper lobe segment)	–	died 3.5 weeks after diagnosis	Angiography	preoperative
ACXPA	PA	Bicuspid aortic valve, aortic stenosis	–	Aortic valve replacement, Ligation LCX	Alive	Angiography	preoperative
ACXPA	RPA	CoA, VSD, ASD	Cystic renal dysplasia, vertebral body malformation	Reimplantation of LCX, patch closure of VSD and ASD, CoA resection	Alive	Angiography	preoperative
ACXPA	RPA	CoA, bicuspid aortic valve,	–	Aortic valve replacement, Reimplantation LCX	Alive	Angiography	preoperative
ASCAPA	PA	–	Diaphragmatic hernia	Reimplantation of SCA, Reoperation due to coronary artery stenosis 3 months after reimplantation	died during reoperation	Angiography	preoperative
ASCAPA	RPA	Shone Complex	–	Patch enlargement of the aortic arch, aortic valve commissurotomy, resection of interatrial septum, patch closure. Reimplantation of SCA	died 6 days after surgery	surgery	intraoperative

*ALCAPA* Anomalous left coronary artery connected to the pulmonary artery, *ARCAPA* Anomalous right coronary artery connected to the pulmonary artery, *ACXPA* Anomalous circumflex coronary artery connected to the pulmonary artery, *ALADPA* Anomalous left anterior descending coronary artery connected to the pulmonary artery, *ASCAPA* Anomalous single coronary artery connected to the pulmonary artery. *CoA* Aortic coarctation, *VSD* Ventricular septal defect, *ASD* Atrial septal defect, *LPSVC* Left persistent superior vena cava, *HLHS* Hypoplastic left heart syndrome, *TGA* Transposition of the great arteries, *PDA* Patent ductus arteriosus

### Associated extracardiac anomalies

In the ALCAPA group, there was one patient with a pelvic kidney, one patient with Rubinstein-Taybi syndrome, and two patients with Scimitar syndrome having hypoplasia of the right lung. In the ARCAPA group, there was one patient with fetal alcohol syndrome, one patient with Goldenhar syndrome, and one patient with VACTERL association with hearing impairment. In the ALADPA group, there were no patients with extracardiac malformations. In the ACXPA group, there was one patient with pulmonary hypoplasia and one with cystic renal dysplasia combined with a vertebral body malformation. In the ASCAPA group, there was one patient with a diaphragmatic hernia. An overview of the extracardiac anomalies for each patient with the associated coronary anomaly is given in Table 2.

Patients with associated cardiac anomalies more frequently have extracardiac malformations (*p* < 0.001) and are more likely to have a connection to the right pulmonary artery (*p* < 0.001) than patients without associated cardiac anomalies, as shown in Table 3.

**Table 3:** Overview of the association between cardiac and extracardiac anomalies with the location of the connection between coronary artery and pulmonary artery

		Without associated cardiac anomalies *n* = 104	With associated cardiac anomalies *n* = 16	
**Associated extracardiac anomalies**	no *n* = 110	102	8	*p* < .001
	yes *n* = 10	2	8	
**Location of the connection between coronary arteries and pulmonary artery**	PA *n* = 107	98	9	*p* < .001
	RPA *n* = 11	3	8	
	LPA *n* = 2	1	1	

#### Clinical Outcomes

Perioperative mortality, defined as any death occurring within 30 days after surgery, occurred in 12 patients. Eight of these 12 patients had ALCAPA without another associated cardiac defect and one patient had associated VSD and CoA. All nine patients had their surgery before 1992. Among the other three patients, who died perioperatively from the later era, two patients had ASCAPA. One of them also had aortic coarctation and died 6 days after the surgery due to coronary ischemia and the other one died on the operating table during the second coronary surgery. One further patient had ALCAPA with aortic coarctation, small left heart structures and Scimitar syndrome. In this patient the diagnosis of ALCAPA was made after the initial coarctation surgery by persisting unstable hemodynamic situation. Even after surgical treatment of ALCAPA and pulmonary veins, the patient died due to circulatory failure.

## Discussion

The present study comprises one of the largest single-center ALCAPA cohorts worldwide, alongside seventeen additional patients with other ACAPA variants, including those with anomalous origins of the right coronary artery, the circumflex artery, or a single coronary artery connecting to the main pulmonary artery or the right or left pulmonary artery. This study evaluated the prevalence of associated congenital heart defects and non-cardiac anomalies in patients with anomalous coronary arteries originating from the pulmonary artery, based on a retrospective analysis of 120 patients treated at our center in the last 50 years.

Aberrant coronary arteries originating from the pulmonary arteries are rare congenital anomalies with significant clinical implications. The most well-known of these is ALCAPA, which constitutes a small fraction of congenital heart defects but carries a high mortality rate if left untreated.

ALCAPA and other ACAPA variants are typically not diagnosed prenatally. Abnormal coronary artery from the pulmonary artery can present clinically after lowering of pulmonary vascular resistances in the first weeks of life with life-threatening cardiogenic shock due to a coronary steal phenomenon responsible for progressive myocardial ischemia. This is the reason, why most patients, especially in the absence of sufficient coronary collateral flow, require surgical repair.

In the presence of further associated cardiac defects, the usual hemodynamic situation is altered depending on the type of associated defect. That makes the already challenging preoperative diagnosis even more complicated. However, the correct diagnosis is of practical importance, since the clinical course can be disastrous in cases of misdiagnosed abnormal coronary artery from the pulmonary artery, in which myocardial ischemia becomes apparent after surgery, often with fatal outcome [10, 11]. Hence, Laux et al. describe compromised postoperative survival in those patients, which is possibly related to the unrecognized diagnosis of a coronary abnormality but also due to midterm complications related to the associated cardiac defect [8]. In our cohort, all three patients with a postoperative diagnosis of ACAPA had other associated congenital heart defect and two of them died within three months after the surgery. This emphasizes the importance of a clearly defined coronary anatomy in patients with complex congenital heart defects prior to initial surgery.

Our study demonstrated that ALCAPA patients, unlike to other ACAPA variants, less frequently present with associated cardiac or extracardiac malformations. Our data demonstrated that 66.7% of ARCAPA patients, 85.7% of ACXPA patients, and 50% of ASCAPA patients had additional cardiac anomalies, compared to only 4.9% of ALCAPA patients. This observation aligns with the literature, which reports a high prevalence of associated congenital heart defects in ARCAPA and ACXPA patients [12–14].

In our cohort, most associated cardiac anomalies in these groups were left-sided obstructive lesions, although the literature describes a variety of congenital heart defects in ALCAPA patients [9, 15, 16]. Interestingly, in our cohort two patients with ALCAPA also had scimitar syndrome. An association of scimitar syndrome with ALCAPA is described in a series by Bo et al. in 3 of 54 patients with Scimitar syndrome, as well as in the study of Flores et al. in 2 of 10 complex ALCAPA patients [15, 17]. Thus, during the diagnosis and potential surgery for congenital heart defects, coronary anomalies should always be considered and explicitly searched for. Patients with additional cardiac defects often, especially those with complex congenital heart defects, such as HLHS, face more complicated surgical interventions and higher perioperative risk.

Associated cardiac defects contributed to poorer perioperative survival. The presence of associated cardiac anomalies significantly impacts the prognosis of patients with coronary artery anomalies, as demonstrated in our study and also described in the recent multicenter study of Flores et al. with 21 centers and 258 ALCAPA patients [15].

In our study, the outcomes varied significantly based on the presence of associated anomalies.

ACAPA variants other than ALCAPA were also often associated with extracardiac anomalies. Extracardiac malformations were more frequent in patients with associated cardiac anomalies. These included a wide range of conditions such as pelvic kidney, Rubinstein-Taybi syndrome, fetal alcohol syndrome, Goldenhar syndrome, and VACTERL association. Notably, patients with ACXPA exhibited complex extracardiac malformations, such as cystic renal dysplasia with vertebral body malformation, highlighting the systemic nature of these congenital syndromes.

## Limitations

Our study provides valuable insights into the prevalence and implications of associated cardiac and extracardiac anomalies in patients with coronary artery anomalies from the pulmonary artery. The main limitation of the study is its retrospective nature, which may introduce biases related to changes in diagnostic techniques and surgical practices over the decades. Moreover, the small sample sizes for non-ALCAPA anomalies limit the generalizability of our findings.

## Conclusion

Thirteen percent of patients with APACA, particularly those other than ALCAPA, exhibit further associated cardiac anomaly. These patients also significantly more frequently show associated extracardiac anomalies. Since associated anomalies have a significant impact on survival, a timely diagnosis is crucial. Particularly in patients with left heart obstructions and a challenging postoperative course as well as in patients with associated extracardiac anomalies, an abnormal coronary artery originating from the pulmonary artery should be considered. This emphasis the importance of a precise diagnosis of coronary anatomy, which is crucial for preoperative planning and the success of the surgery.

## Conflict of interests

The authors declare no competing interests.

## Data Availability

No datasets were generated or analysed during the current study.
